# The Characterization of Binding between Aptamer and Bisphenol A and Developing Electrochemical Aptasensors for Bisphenol A with Rationally Engineered Aptamers

**DOI:** 10.3390/bios12110913

**Published:** 2022-10-23

**Authors:** Liying Liu, Hao Yu, Qiang Zhao

**Affiliations:** 1 State Key Laboratory of Environmental Chemistry and Ecotoxicology, Research Center for Eco-Environmental Sciences, Chinese Academy of Sciences, Beijing 100085, China; 2 University of Chinese Academy of Sciences, Beijing 100049, China; 3 School of Environment, Hangzhou Institute for Advanced Study, UCAS, Hangzhou 310024, China

**Keywords:** bisphenol A, aptamer, ITC, electrochemical sensor, binding affinity

## Abstract

Bisphenol A (BPA) is widely used in the manufacture of polycarbonate and epoxy-resin-based products, and BPA contamination often happens in a variety of types of environment and food stuffs. BPA can cause many harmful effects to health due to its high toxicity. The rapid detection of BPA is of great significance in environmental monitoring and food safety. Nucleic acid aptamers show advantages in biosensors due to good chemical stability, the ease of labeling functional groups, and target binding that induces conformation change. Here, we performed a thorough characterization of the binding performance of one 60-nt anti-BPA DNA aptamer with isothermal titration calorimetry (ITC). We found the crucial region of the aptamer sequence for affinity binding with BPA, and the aptamer was able to be truncated to 29-nt DNA without losing affinity. We then developed a simple reagent-less electrochemical aptamer-based sensor for rapid BPA detection with this engineered aptamer. The truncated aptamer with a redox tag methylene blue (MB) was immobilized on a gold electrode. BPA-binding induced the conformation change of the MB-labeled aptamer, moving the MB close to the electrode surface and causing a significant current increase in MB in square wave voltammetry (SWV). Under optimized conditions, we achieved the quantitative detection of BPA with a detection limit of BPA at 0.1 μM. This sensor showed quick response to BPA and could be regenerated by washing with deionized water. This sensor was selective, and it allowed detecting BPA in complex samples, showing its potential in practice. This study will help in further applications of the aptamers of BPA.

## 1. Introduction 

Bisphenol A (BPA) is a vital raw material in the production of epoxy resins and polycarbonate plastics in the industry, and BPA-related products have been widely applied in many fields, such as food packing, construction material, electronic product, etc. [1,2]. During the production and consumption of products, BPA leach into many types of environment, such as drinking water, waste water, soil, and food [1,2]. BPA contaminations often happen due to the intense use of BPA [3,4]. BPA exposure to humans can cause many harmful effects on human health, such as endocrine disrupting, reproductive disorders, cancer risks, immunosystem dysfunction, genotoxic effects, obesity, diabetes, cardiovascular diseases, etc. [5,6,7,8]. Due to the high toxicity of BPA, BPA detection is of importance for environmental monitoring, food safety, risk assessment, and toxicology studies [9,10,11,12]. The usual methods for BPA detection include high-performance liquid chromatography (HPLC), HPLC-mass spectrometry (MS), gas chromatography (GC)-MS, enzyme-based assays, immunoassays, etc. [9,10,11,12]. The LC-MS and GC-MS may suffer from high costs, require professional personnel, and time-consuming sample treatments. The immunosensors and immunoassays require antibodies, and antibodies meet some limitations in stability and preparation. By contrast, biosensors for BPA have strength in portability and rapidity, and they are suitable for the on-site rapid detection of BPA, attracting increasing attention [10,13,14]. 

Aptamers are single-stranded DNA or RNA selected from a random sequence of nucleic acid library, which can specifically bind to targets with high affinity [15,16]. The aptamers show advantages such as easy preparation, high stability, and modifications with functional groups. Aptamers are promising in many applications, such as drug discovery, affinity separation and delivery, therapeutics, biosensors, and diagnostics [17,18,19,20]. The aptamer-based assays and sensors attracted increasing attention, circumventing some limitations of antibody-based methods in antibody preparation and antibody stability. Due to the unique feature of aptamers, aptamer assays and sensors with versatile formats have been reported, and many of them cannot be achieved by using immunoantibodies [19,20,21,22]. 

Aptamers of BPA were firstly reported by in 2001 [23]. Since the discovery of DNA aptamers for BPA, aptamer-based methods emerged for BPA detection with different formats [11,22,24,25]. Affinity binding is the basis of the aptamer applications, so the aptamer characterization is an important task, which will also be helpful in further aptamer design and engineering [26,27,28]. However, the characterization of affinity binding between aptamer and BPA is still relatively limited, especially with respect to the characterization with isothermal titration calorimetry (ITC), a widely used label-free technique for molecular interaction with high reliability [26,27]. The binding region of aptamer and the crucial aptamer sequence for BPA binding remain unclear. Aptamer-based sensors and assays for BPA included colorimetric assay, fluorescence assay, electrochemical sensors, etc. [22,24,25,29]. Electrochemical sensors are suitable for on-site detection. They show advantages such as low costs, rapid responses, and reusability. Some aptamer-based electrochemical sensors have been developed [24,25]; however, they may still need tedious steps and additional reagents for BPA sensing and cannot be reused. The aptamer electrochemical sensors relying on binding-induced conformation change are simple reagent-less electrochemical sensors with increasing attention [30,31,32].

In this study, we applied isothermal titration calorimetry to fully study BPA-aptamer binding. We found a 60-mer DNA aptamer showed affinity to BPA with a *K*_d_ of about 16 μM, while another 63-mer DNA aptamer did not show binding in ITC. We made serial truncations of the 60-mer DNA aptamers and identified the crucial aptamer sequence for BPA binding, And a truncated short 29-mer aptamer still showed a *K*_d_ of about 10 μM. Based on the affinity characterization, we further developed a simple aptamer electrochemical sensor for BPA by using the rationally designed sequence that showed binding-induced structure change. This aptamer electrochemical sensor allowed the rapid detection of BPA, with a detection limit of 0.1 μM. This study will facilitate the application of the aptamer of BPA.

## 2. Materials and methods

### 2.1. Chemicals and Materials 

Bisphenol A (BPA), ampicillin (Amp), tyrosine (Tyr), tetracycline (Tet), arginine (Arg), 6-mercaptohexanol (MCH), and tris-(2-carboxyrthyl) phosphine hydrochloride (TCEP) were purchased from Sigma. 4,4′-Dihydroxybiphenyl (BP) and bisphenol S (BPS) were purchased from J&K Chemical (Beijing, China). Oxytetracycline (Oxy) and synthesized DNA oligonucleotides were ordered from Sangon Biotech (Shanghai, China). Reagents with analytical grade were used in all experiments. Solutions in experiments were prepared by ultrapure water from Elga Labwater system (Purelab Ultra Genetic Type, Lane End, UK). 

### 2.2. Isothermal Titration Calorimetry Measurement 

Isothermal titration calorimetry (ITC) analyses were conducted with instrument MicroCal PEAQ-ITC (Malvern). Typically, BPA solution and aptamers solution were prepared in a binding buffer (25 mM Tris-HCl (pH = 8.0), 100 mM NaCl, 25 mM KCl, 10 mM MgCl_2_, and 2% dimethyl sulfoxide (DMSO)). To determine the affinity of aptamers with ITC, the BPA solution (500 μM, 60 μL) in an injection syringe was gradually titrated into a solution with 30 μM aptamers (270 μL) in a sample cell. The syringe stirred at a speed of 800 rpm, and the reference power was fixed at 8 μcal/s. Following an initial 600 s equilibrium step, standard titrations began with first 0.4 μL injection and 19 successive 2.0 μL injections every 120 s. We conducted a blank experiment by a direct titration of the BPA solution into the binding buffer without aptamers. For ITC analysis, the integrated heat pulse area of each titration was plotted with the molar ratio of the BPA to aptamers after a subtraction of blank data. The determined dissociation constants (*K*_d_s) were finally obtained by the packaged data analysis software of the instrument.

### 2.3. Preparation of Aptamer Modified Electrode

To prepare the aptamer-modified electrode, we first polished gold electrodes with a 2 mm diameter (CH Instrument Co., Shanghai, China) on a microcloth with 0.05 μm alumina slurries. After polishing, the gold electrode was cleaned by ultrasonication with water, ethanol, and water. Then, the electrode was cleaned by an electrochemical procedure according to the same steps described in the previous report [32]. 

To immobilize the aptamer on the cleaned gold electrode, thiolated aptamers with methylene blue (MB) labels reacted with 1 mM TCEP in 1 × PBS solution (137 mM NaCl, 2.7 mM KCl, 10 mM Na_2_HPO_4_ and 2 mM KH_2_PO_4_) (pH 7.5) for 1 h at 4 °C. We then placed the gold electrode in 1 × PBS (pH 7.5) solution with an activated aptamer (1 μM) for 1 h. After that, the aptamer-modified gold electrode was placed in 1 × PBS (pH 7.5) solution containing 2 mM MCH for 2 h to passivate the gold electrode’s surface. After MCH blocking, the aptamer-modified gold electrode was washed and ready for use.

### 2.4. BPA Detection by Electrochemical Aptasensor

An electrochemical workstation (CHI 660E, CH Instrument Co., Shanghai, China) was employed for experiments. Electrochemical measurements were made with a conventional three-electrode system, including the aptamer-modified gold electrode (2 mm diameter) as a working electrode, the platinum wire counter electrode, and the Ag/AgCl (3 M KCl) reference electrode. The aptamer-modified electrode was incubated with varying concentrations of BPA for 3 min in a binding buffer (25 mM Tris-HCl (pH, 8.0), 100 mM NaCl, 25 mM KCl, and 10 mM MgCl_2_). Then, square wave voltammetry (SWV) analysis was performed with a scanning range from 0 to −0.5 V, step potential of 1 mV, frequency of 300 Hz, and amplitude of 25 mV. We measured the peak current of MB in SWV for BPA detection. Each measurement was repeated at least three times. After sensing, the electrode was regenerated by washing with deionized water to remove the bound BPA.

## 3. Results and Discussions

### 3.1. Characterization of Aptamer–BPA Binding with ITC

The aptamers of BPA were first reported by Jo al [23], and a variety of methods for detecting BPA based on the aptamers have been developed [11,22,24,25]. However, the characterization and verification of the affinity of aptamers of BPA remain limited. Isothermal titration calorimetry (ITC) is a commonly used method for affinity binding, and the ITC characterization of BPA–aptamer binding has not been reported yet. Here, we used ITC to characterize the affinity of aptamers with BPA as ITC is a reliable label-free method for molecular interaction studies [26,27]. The reported 60-mer aptamer (BP60: 5′-GTT GGG CAC GTG TTG TCT CTC TGT GTC TCG TGC CCT TCG CTA GGC CCA CA-3′) [23] without a label was applied in the ITC study.

**Figure 1:**
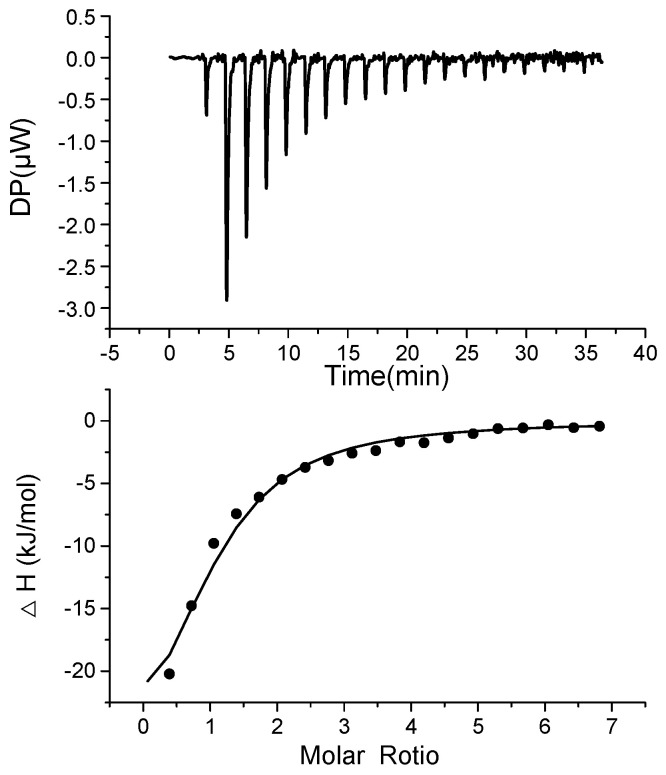
ITC measurement (**top** panel) and binding isotherm fitted by one set of the site’s model (**bottom** panel) for the titration of BPA to aptamer BP60.

As shown in Figure 1, when the aptamer bound to BPA, heat was released, which was measured by the calorimeter during the gradual titration of BPA into a BP60 sample solution in the ITC cell. The dissociation constant (*K*_d_) of BP60 to BPA was determined to be 15.8 ± 2.0 μM by ITC, confirming that BP60 can bind with BPA. In contrast, the other reported 63-mer aptamer (BP63: 5′ -CCG GTG GGT GGT CAG GTG GGA TAG CGT TCC GCG TAT GGC CCA GCG CAT CAC GGG TTC GCA CCA-3′) [23] did not show binding with BPA in ITC analyses (Figure S1 in Supporting Information), and the reason was unknown.

**Figure 2:**
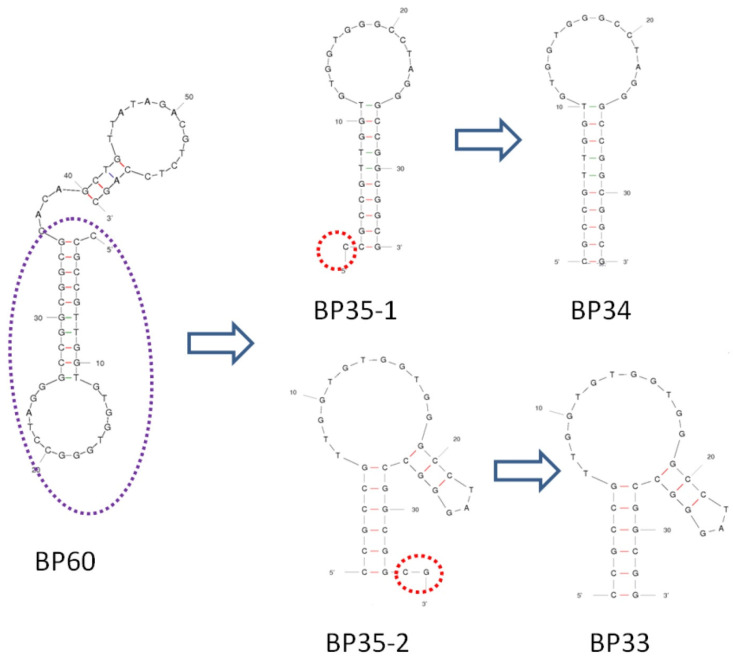
The secondary structures of BP60, BP35 (BP35-1 and BP35-2), BP-34, and BP-33.

Figure 2 shows the predicted secondary structure of aptamer BP60 according to Mfold [33]. In order to identify the key sequence of BP60 for affinity recognition and to obtain a short sequence of aptamer, we made serial truncations in the sequence of BP60 and tested the truncated aptamers with ITC analysis. We serially cut five bases from the 3′ end of the original sequence BP60 and obtained BP55, BP50, BP45, BP40, and BP35 (Table 1). The sequences of the original and truncated aptamers are shown in Table 1, and their affinities to BPA were determined by ITC analyses (Figure S2). The *K*_d_s of BP60, BP55, BP50, BP45, BP40, and BP35 ranged from 9 μM to 20 μM (Table 1), indicating that 25 nucleotides can be truncated from the 3′ end of BP60 without greatly reducing aptamer affinities. It is predicted that BP35 has a simple stem-loop structure (Figure 2). The 35-mer aptamer BP35 may have two different secondary structures, shown as BP35-1 and BP35-2 in Figure 2. In order to determine which was the possible secondary structure of BP35 responsible for BPA binding, we truncated the C from the 5′ end of BP35-1 to obtain BP34 and removed the C and G at the 3′end of BP35-2 to obtain BP33, respectively. BP34 showed a *K*_d_ of 307 ± 249 μM, indicating that the C close to 5′ end at BP35 is crucial in the binding of the BPA and aptamer. The *K*_d_ of BP33, truncated from BP35-2, was 10.5 ± 1.5 μM, which is similar to that of BP60. Therefore, it is likely that the secondary structure of BP35-2 (Figure 2) is responsible for binding BPA. This result indicates that BP35 can be further truncated into a 33-mer aptamer BP33 that retains affinity to BPA.

**Figure 3:**
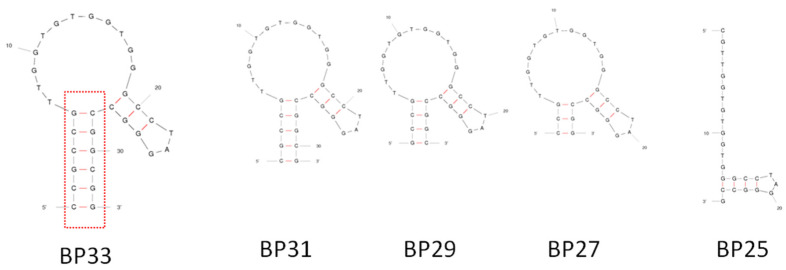
Predicted secondary structures of BP33 and its truncated aptamers.

BP33 has a simple stem-loop structure (Figure 2 and Figure 3), and the stem contains six pairs of complementary bases. In order to further identify the crucial sequence of the aptamer in affinity recognition, we further serially truncated the complementary bases of the stem and measured the affinity of the obtained sequences of BP31, BP29, BP27, and BP25 by ITC (Table 1 and Figure S3). The *K*_d_s of BP31 and BP29 to BPA were 13.4 ± 1.5 μM and 12.4 ± 3.4 μM, respectively, which were similar to the affinity of BP33. BP27 had an increased *K*_d_ of 56 ± 9.0 μM, indicating that binding affinity of BP27 was significantly reduced. After truncating another pair of complementary bases of BP27, BP25 cannot form a stem-loop structure according to the prediction, and ITC analyses confirmed that BP25 did not bind with BPA. Clearly, the original sequence BP60 can be truncated to a short-sequence BP29, maintaining a high affinity to BPA. The results indicate that the stem stability is important for aptamer affinity and a stable stem with proper length is required for the aptamer to form a stable stem-loop structure and maintain strong binding affinities to BPA. 

### 3.2. Identification of Crucial Bases on Aptamers for BPA Binding

In order to gain improved insights into affinity binding between BPA and aptamer, we further investigated the mutants of BP33 using ITC. The tested mutants involved single G to T, C to A, T to A, and A to T substitutions of all bases in the loop region and some of G bases in the stem region (Table S1). 

When the G bases in the loop region of BP33 mutated to T bases, most mutant sequences lost the binding to BPA, with the exception of BP33-19GT. The *K*_d_s of BP33-19GT increased to 151 ± 16.8 μM, showing that the affinity to BPA of the mutants significantly decreased. Clearly, all G bases on the loop region are highly conserved and important for the aptamer to have high binding affinities. The *K*_d_s of BP33-22TA, BP33-20CA, BP33-21CA, and BP33-27CA were close to that of BP33, indicating that the single mutation at these positions does not affect the aptamer’s affinity. BP33-11TA, BP33-13TA, and BP33-16TA with substitutions T to A in the loop region of BP33 showed the *K*_d_s between 22 and 24 μM, suggesting these mutants maintained their binding affinity to BPA. The binding affinity of BP33-7TA to BPA greatly decreased (*K*_d_s = 68.4 ± 4.9 μM), and BP33-8TA and BP33-23AT did not show binding to BPA. This confirms that the 7T, 8T, and 23A bases of aptamers were conserved and important in the affinity recognition of BPA.

The substitution of G to T in the stem region of BP33 also caused a loss or reduction in binding affinity to BPA as the mutation reduced the stem’s stability. The BP33-stem-30GT still showed binding with BPA in ITC analyses, but it had a weaker binding affinity to BPA (*K*_d_ = 51.2 ± 10.4 μM). The reason for the significant decrease in affinity may be that the original complementary base pairs, which were close to the loop, were destroyed after the mutation of the G base, and the stability of the stem-loop structure also changed. The results indicate that the complementary bases near the loop play an important role in stabilizing the stem-loop structure. The results also demonstrate that the loop region of the aptamer is the possible binding site of BPA on the aptamer. 

### 3.3. Aptamer-Based Electrochemical Switch Sensor for BPA Detection

**Figure 4:**
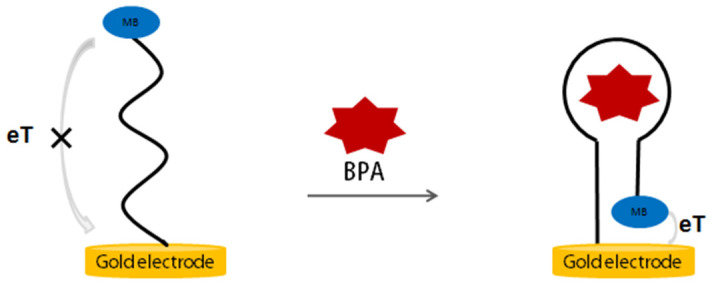
The principle of aptamer-based electrochemical switch sensors for BPA detection.

After determining the aptamer affinity and identifying the crucial region of the aptamer sequence, we reported a simple aptamer electrochemical sensor for BPA by rationally designing the aptamer sequence. Figure 4 shows the principle of the electrochemical aptasensor for the detection of BPA. The anti-BPA aptamer having a redox tag methylene blue (MB) label is immobilized on a gold electrode via gold–sulfur chemistry. In the absence of BPA, the aptamer has an open structure, and the MB labeled on the aptamer is relatively distant from the gold electrode’s surface, causing a small peak current of MB due to the weak electron transfer efficiency between MB and the electrode’s surface. When BPA is present, BPA binding induced a conformation change, and a stable structure is formed, causing the MB close to the electrode surface and a significant increase in the current of MB in square wave analysis (SWV). Therefore, the quantitative detection of BPA can be achieved by measuring the increase in current signal.

**Figure 5:**
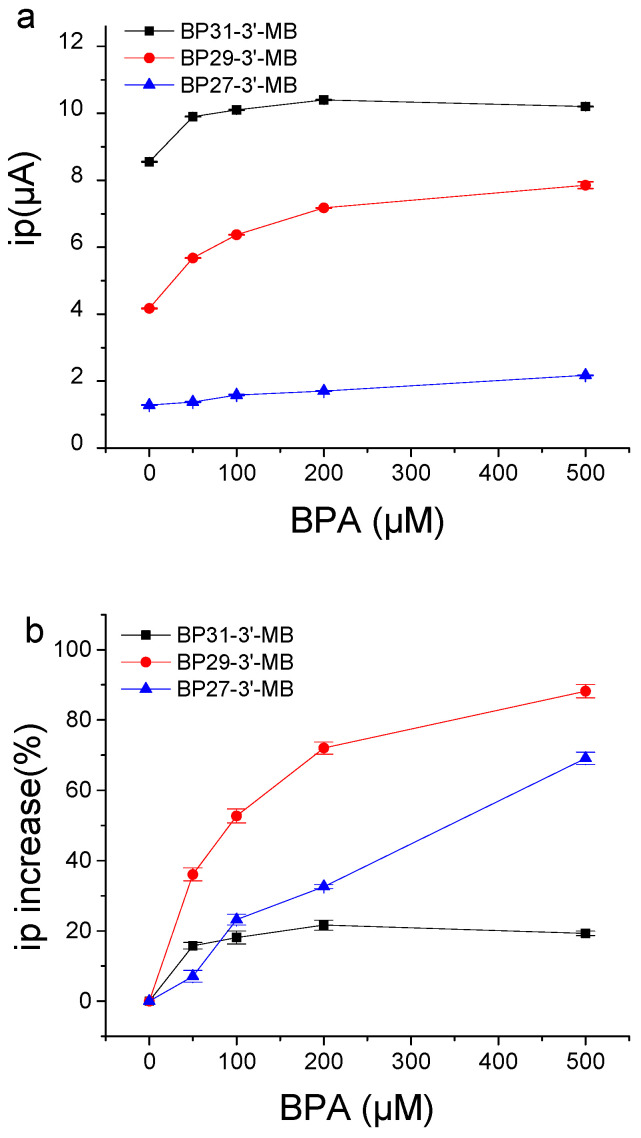
Responses of BP27-3′-MB-modified electrode, BP29-3′-MB-modified electrode, and BP31-3′-MB-modified electrode with respect to BPA. (**a**) Peak current (ip) values versus BPA concentrations. (**b**) Comparison of different aptamer-modified electrodes in the increased percentage in peak currents caused by BPA. The binding buffer contained 25 mM Tris-HCl (pH = 8.0), 100 mM NaCl, 25 mM KCl, and 10 mM MgCl_2_. Frequency was set as 250 Hz in SWV.

We designed three MB-labeled aptamers (BP27-3′-MB, BP29-3′-MB, and BP31-3′MB) with different lengths of stem, and they had a thiol group at the 5 ‘end and MB label at the 3′end (Table S2). We prepared the aptamer-modified gold electrode to test the performance of electrochemical aptasensors constructed by BP27-3′-MB, BP29-3′-MB, or BP31-3′MB. Figure 5 shows the current change of the electrochemical aptasensors in response to BPA binding. The initial peak current ip values without BPA (ip_blank._) gradually increased with the increase in complementary base pairs in the stem. The BP31-3′MB modified electrode produced a high ip_blank_, while the BP27-3′MB-modified electrode produced a low ip_blank_. This is because BP31-3′-MB has longer complementary base pairs in the stem and can form a more stable stem-loop structure in the absence of BPA, resulting in closer distances between MB to the electrode’s surfaces and the higher peak current signal. BP27 had an unstable stem and open structure in the absence of BPA; thus, MB was far from the electrode surface, producing low current signals.

All tested sensors produced increased currents in response to BPA addition (Figure 5A). The BP29-3′-MB-modified electrode showed higher responses to BPA, and the peak current increased by 88% upon the addition of 500 μM BPA (Figure 5B). The BP27-3′-MB-modified electrode produced a small current increase with respect to BPA, which is possibly due to the weak affinity of BP27 (Table 1). BP31 had a more stable stem-loop structure, and BPA-binding induced less conformation changes; thus, the BP31-3′-MB-modified electrode showed smaller current changes in response to BPA, with only a 19% current increase upon the addition of 500 μM BPA (Figure 5B). Therefore, the BP29-3′-MB-modified electrode was used to detect BPA with higher sensitivities.

After the detection of BPA, the electrochemical aptasensor regenerated by simple washing with ultrapure water. As shown in Figure S4, the ip value was close to the ip_blank_ after washing with ultrapure water, meaning that BPA dissociated from the electrode by washing. This can be attributed to the fact that the aptamer’s affinity binding requires metal ions in the buffer, and water without the presence of metal ions causes the aptamer lose affinity. After cycles of regeneration processes, the BP29-3′-MB-modified electrode still had a good response to BPA. The result indicates that this electrochemical aptasensor can be reused.

To obtain more sensitive electrochemical detections for BPA, we investigated the influence of some experimental conditions, including the concentration of MgCl_2_ in the binding buffer and the frequency of SWV. The MgCl_2_ in the binding buffer affects the affinity binding between BPA and the aptamers and the stability of the stem-loop structure of the aptamers. As shown in Figure S5, in the absence of BPA, ip_blank_ increased with the increase in MgCl_2_, because MgCl_2_ helps the hybridization of complementary bases of the stem in aptamers and enhances the stability of stem, which leads to the MB label on the aptamer close to the electrode’s surface and causes the increase in ip_blank_. In the presence of BPA, the ip change caused by BPA additions firstly increased with the increase in MgCl_2_ concentrations until 10 mM MgCl_2_ was applied, indicating that a certain concentration of MgCl_2_ was favorable for the formation of proper secondary structure, aptamer affinity, and the detection of BPA. The addition of 10 mM MgCl_2_ in the binding buffer solution was applied to further experiments. 

**Figure 6:**
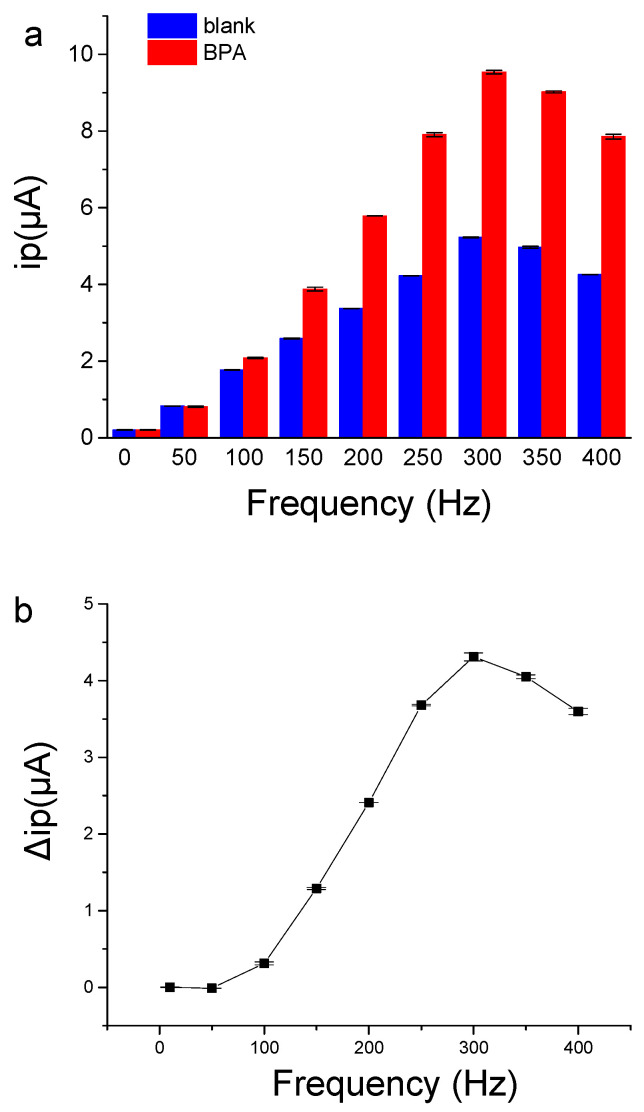
The effects of frequency on current signals of BP29-3′-MB electrode. (**a**) The peak current value of BP29-3′-MB electrode in the absence or the presence of 500 μΜ BPA. (**b**) The peak current signals change caused by 500 μΜ BPA. The binding buffer contained 25 mM Tris-HCl (pH = 8.0), 100 mM NaCl, 25 mM KCl, and 10 mM MgCl_2_.

The frequency of SWV had great influence on the sensing performance of the aptamer sensor. As shown in Figure 6, when the frequency was low, the current signal was weak, and the ip change caused by BPA was also small. When the frequency increased to 300 Hz, the peak current and the ip change caused by BPA binding were the largest. The results demonstrate that the frequency of SWV is a key factor for aptamer electrochemical sensors, which is consistent with a previous report [32]. Therefore, the frequency of 300 Hz in SWV was selected for the electrochemical detection of BPA to achieve more sensitive signal-on responses to BPA binding.

**Figure 7:**
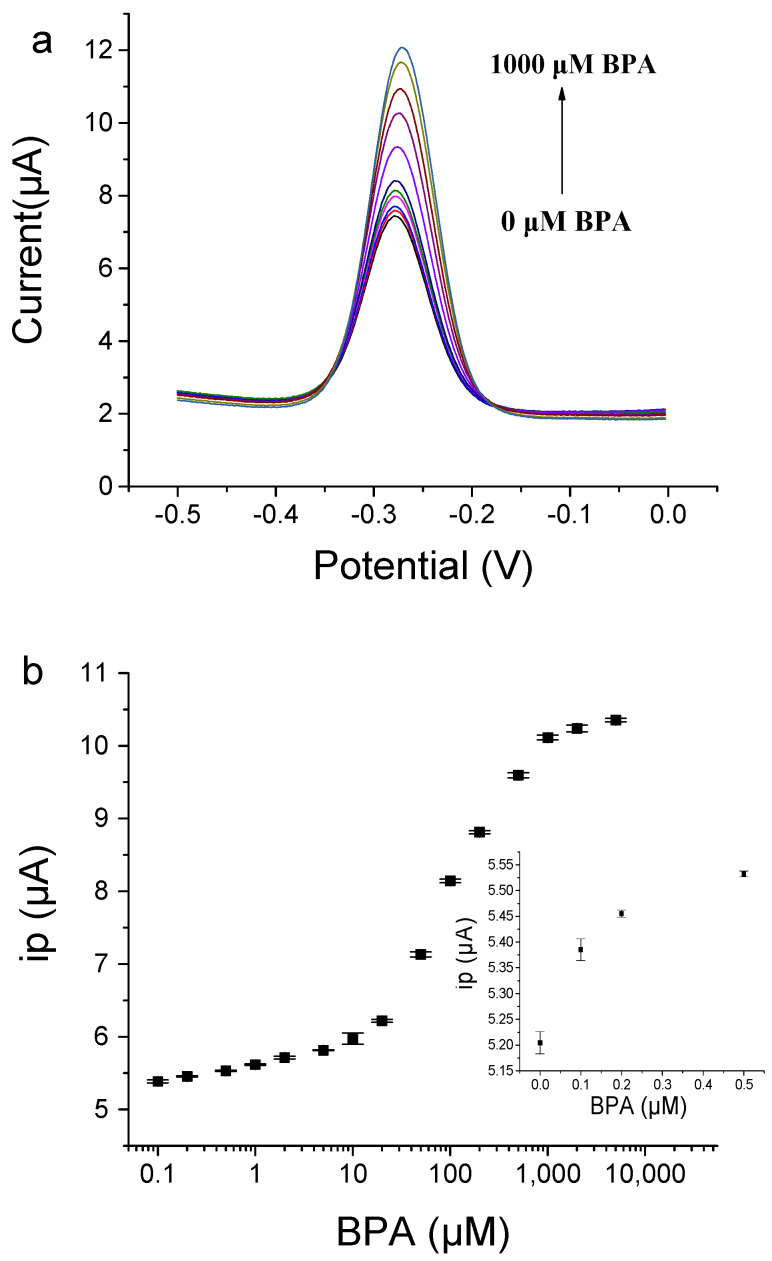
The aptamer-based electrochemical sensor for BPA detection by using BP29-3′-MB modified electrode. (**a**) SWV signals of various concentrations of BPA. (**b**) Plot of peak current responses towards BPA concentration. The inset shows the peak current responses to low concentrations of BPA and blank samples.

Under optimized conditions, we successfully detected various concentrations of BPA by using the BP29-3′-MB-modified electrode. With the addition of BPA, the peak current value gradually increased (Figure 7). BPA in the concentration range from 0.1 μM to 1000 μM was detected. For the low concentration range from 0.1 μM to 10 μM, the linear fitting equation was Y = 0.2838 logX + 5.6418 (R² = 0.9794), where Y was the peak current and X was the concentration of BPA. For concentration ranges from 10 μM to 1000 μM, the linear fitting equation was Y = 2.2177 logX + 3.5639 (R² = 0.9883). The two linear relationships may be caused by two binding affinities of the immobilized aptamers on the gold electrode, but the exact reason is not known. The immobilization of the aptamer on the electrode may have enhanced its affinity due to high local concentrations and molecular crowding [34,35]. The limit of detection (LOD) for BPA was determined to be 0.1 μM based on the fact that the signal change was three times that of the standard deviation of the blank sample signal. Compared with other methods (Table S3) [11,14,22,24,25,36,37,38,39,40,41], our method shows a medium sensitivity, because this aptamer has binding affinities with *K*_d_ at the μM level and this aptasensor does not involve a signal amplification strategy. However, our electrochemical aptasensor shows advantages such as simple operation and rapid analysis. In addition, this aptasensor can be reused with good regeneration by washing the gold electrode with water. The electrochemical aptasensor still showed good responses to BPA after the storage of the aptamer-modified gold electrode in a binding buffer at 4 °C for five days (Figure S6). This result demonstrates that the electrochemical aptasensor had good stability.

We assessed the selectivity of the electrochemical aptasensor for BPA by testing analogues of BPA, including BPA analogs (BP and BPS), tyrosine (Tyr), arginine (Arg), Oxytetracycline (Oxy), ampicillin (Amp), and tetracycline (Tet). All tested small molecules did not induce remarkable ip changes (Figure S7). The simultaneous presence of the tested small molecules did not affect the detection of BPA. The results indicate that method exhibits good selectivity in analyzing BPA.

The performance of our electrochemical aptasensor in actual water samples was evaluated by testing the ip responses to BPA in 5-fold diluted tap water and bottled water. As displayed in Figure S8, the electrochemical aptasensor responded well to the BPA over the range of 0.1–1000 μM in actual water samples. The results demonstrate the feasibility of the electrochemical aptasensor for the detection of BPA in complex samples, and the sensor has potential for real sample analysis.

## 4. Conclusions

In summary, we performed a full characterization of the affinity performance of anti-BPA aptamers by using ITC and developed an aptamer-based electrochemical switch sensor for the detection of BPA according to the identified crucial structure of aptamer. We verified that the dissociation constant of the aptamer of BPA was at μM levels. After testing a series of the truncated and mutant aptamer sequences, we found that a short 29-mer aptamer possessing a simple crucial stem-loop structure still possessed high binding affinities. With the short, truncated aptamer, we developed an electrochemical aptasensor for BPA detection. The BPA binding induced a conformation change in the aptamer, moving the MB close to the electrode surface and the current of MB increase in SWV. The detection limit of BPA reached 0.1 μM. The operation of this reagent-less aptamer electrochemical method is easy. The aptamer-modified electrode can be regenerated by washing with deionized water and reused. This sensor shows its potential in practice, and this study is helpful for the application of aptamers of BPA.

## Figures and Tables

**Table 1:** Summary of sequences and binding affinity of truncated aptamers from BP60.

Name	Sequences	*K*_d_/μM
BP60	5′-CCGCCGTTGGTGTGGTGGGCCTAGGGCCGGCGGCGCACAGCTGTTATAGACGTCTCCAGC-3′	15.8 ± 2.0
BP55	5′-CCGCCGTTGGTGTGGTGGGCCTAGGGCCGGCGGCGCACAGCTGTTATAGACGTCT-3′	11.6 ± 1.0
BP50	5′-CCGCCGTTGGTGTGGTGGGCCTAGGGCCGGCGGCGCACAGCTGTTATAGA-3′	9.5 ± 0.5
BP45	5′-CCGCCGTTGGTGTGGTGGGCCTAGGGCCGGCGGCGCACAGCTGTT-3′	12.5 ± 0.8
BP40	5′-CCGCCGTTGGTGTGGTGGGCCTAGGGCCGGCGGCGCACAG-3′	15.4 ± 0.9
BP35	5′-CCGCCGTTGGTGTGGTGGGCCTAGGGCCGGCGGCG -3′	20.2 ± 1.0
BP34	5′-CGCCGTTGGTGTGGTGGGCCTAGGGCCGGCGGCG-3′	307 ± 249
BP33	5′-CCGCCGTTGGTGTGGTGGGCCTAGGGCCGGCGG -3′	10.5 ± 1.5
BP31	5′-CG CCG TTG GTG TGG TGG GCC TAG GGC CGG CG -3′	13.4 ± 1.5
BP29	5′-G CCG TTG GTG TGG TGG GCC TAG GGC CGG C -3′	12.4 ± 3.4
BP27	5′-CCG TTG GTG TGG TGG GCC TAG GGC CGG -3′	56 ± 9.0
BP25	5′-CG TTG GTG TGG TGG GCC TAG GGC CG -3′	NB

NB means no binding.

## Data Availability

Not applicable.

## Supplementary Materials

The following supporting information can be downloaded at: https://www.mdpi.com/article/10.3390/bios12110913/s1, Figure S1: ITC analysis of binding between BPA and aptamer BP63; Figure S2: ITC analysis of binding affinities of BP60 and its truncated aptamers to BPA; Figure S3: ITC analysis affinities of BP33 and truncated aptamers from BP33 to BPA; Figure S4: Effects of regeneration times on responses of the BP29-3′-MB electrode in SWV; Figure S5: Effects of MgCl_2_ on current signals of BP29-3′MB electrode in SWV in the absence and in the presence of BPA; Figure S6: Stability test of responses of BP29-3′-MB modified electrode in SWV; Figure S7: Selectivity test for BPA detection. Figure S8: Detection of BPA spiked in 5-fold diluted tap water and 5-fold diluted bottle water; Table S1: Summary of binding affinity of mutated BP33 probes; Table S2: Sequences of aptamers for fabrication of electrochemical sensors; Table S3: A comparison of some electrochemical aptasensors for BPA.
