# Supplementary material for: The Characterization of Binding between Aptamer and Bisphenol A and Developing Electrochemical Aptasensors for Bisphenol A with Rationally Engineered Aptamers
Source: Biosensors (Basel). 2022 Oct 23;12(11):913. doi: 10.3390/bios12110913 (PMC9688307; doi:10.3390/bios12110913)
Supplement: Supplementary file 1 [file biosensors-12-00913-s001.zip › biosensors-1966831-supplementary.pdf]

## Supporting Information

# The Characterization of Binding between Aptamer and Bisphenol A and Developing Electrochemical Aptasensors for Bisphenol A with Rationally Engineered Aptamers

**Liying Liu [1,2], Hao Yu [1,2] and Qiang Zhao [1,2,3,*]**

[1] State Key Laboratory of Environmental Chemistry and Ecotoxicology, Research Center for Eco-Environmental Sciences, Chinese Academy of Sciences, Beijing 100085, China; liu.liying@craes.org.cn (L.L.); haoyu_st@rcees.ac.cn (H.Y.)
[2] University of Chinese Academy of Sciences, Beijing 100049, China
[3] School of Environment, Hangzhou Institute for Advanced Study, UCAS, Hangzhou 310024, China
[*] Correspondence: qiangzhao@rcees.ac.cn

**Table S1.** Summary of binding affinity of mutated BP33 probes

| Name | Sequences | $K_d$/μM |
|---|---|---|
| BP33-9GT | 5'-CCGCCGTTTGTGTGGTGGGCCTAGGGCCGGCGG-3' | NB |
| BP33-10GT | 5'-CCGCCGTTGTTGTGGTGGGCCTAGGGCCGGCGG-3' | NB |
| BP33-12GT | 5'-CCGCCGTTGGTTTGGTGGGCCTAGGGCCGGCGG-3' | NB |
| BP33-14GT | 5'-CCGCCGTTGGTGTTGTGGGCCTAGGGCCGGCGG-3' | NB |
| BP33-15GT | 5'-CCGCCGTTGGTGTGTTGGGCCTAGGGCCGGCGG-3' | NB |
| BP33-17GT | 5'-CCGCCGTTGGTGTGGTTGGCCTAGGGCCGGCGG-3' | NB |
| BP33-18GT | 5'-CCGCCGTTGGTGTGGTGTGCCTAGGGCCGGCGG-3' | NB |
| BP33-19GT | 5'-CCGCCGTTGGTGTGGTGGTCCTAGGGCCGGCGG-3' | NB |
| BP33-24GT | 5'-CCGCCGTTGGTGTGGTGGGCCTATGGCCGGCGG-3' | 151±16.8 |
| BP33-25GT | 5'-CCGCCGTTGGTGTGGTGGGCCTAGTGCCGGCGG-3' | NB |
| BP33-26GT | 5'-CCGCCGTTGGTGTGGTGGGCCTAGGTCCGGCGG-3' | NB |
| BP33-stem-6GT | 5'-CCGCCTTTGGTGTGGTGGGCCTAGGGCCGGCGG-3' | NB |
| BP33-stem-29GT | 5'-CCGCCGTTGGTGTGGTGGGCCTAGGGCCTGCGG-3' | NB |
| BP33-stem-30GT | 5'-CCGCCGTTGGTGTGGTGGGCCTAGGGCCGTCGG-3' | 51.2±10.4 |
| BP33-7TA | 5'-CCGCCGATGGTGTGGTGGGCCTAGGGCCGGCGG-3' | 68.4±4.9 |
| BP33-8TA | 5'-CCGCCGTAGGTGTGGTGGGCCTAGGGCCGGCGG-3' | NB |
| BP33-11TA | 5'-CCGCCGTTGGAGTGGTGGGCCTAGGGCCGGCGG-3' | 24.4±1.8 |
| BP33-13TA | 5'-CCGCCGTTGGTGAGGTGGGCCTAGGGCCGGCGG-3' | 22.6±6.9 |
| BP33-16TA | 5'-CCGCCGTTGGTGTGGAGGGCCTAGGGCCGGCGG-3' | 24.4±1.6 |
| BP33-22TA | 5'-CCGCCGTTGGTGTGGTGGGCCAAGGGCCGGCGG-3' | 15.2±0.9 |
| BP33-20CA | 5'-CCGCCGTTGGTGTGGTGGGACTAGGGCCGGCGG-3' | 14.6±0.9 |
| BP33-21CA | 5'-CCGCCGTTGGTGTGGTGGGCATAGGGCCGGCGG-3' | 10.5±0.7 |
| BP33-27CA | 5'-CCGCCGTTGGTGTGGTGGGCCTAGGGACGGCGG-3' | 16.0±2.2 |
| BP33-23AT | 5'-CCGCCGTTGGTGTGGTGGGCCTTGGGCCGGCGG-3' | NB |

NB means no binding. The mutated bases are shown in red and underline format.

**Table S2.** Sequences of aptamers for fabrication of electrochemical sensors.

| Name | Sequences |
|---|---|
| BP31-3'-MB | 5'-SH-$(CH_2)_6$-CG CCG TTG GTG TGG TGG GCC TAG GGC CGG CG -MB-3’ |
| BP29-3'-MB | 5'-SH-$(CH_2)_6$-G CCG TTG GTG TGG TGG GCC TAG GGC CGG C -MB-3’ |
| BP27-3'-MB | 5'-SH-$(CH_2)_6$-CCG TTG GTG TGG TGG GCC TAG GGC CGG -MB-3’ |

**Table S3.** A comparison of some electrochemical aptasensors for BPA.

| Strategies | Fabrication time | Analysis time | Dynamic range | LOD | Ref. |
|---|---|---|---|---|---|
| Molybdenum (IV) sulfide nanosheet-based electrochemical impedance spectroscopy (EIS) aptasensor | 18 h | 25 min | 1 nM -10 μM | 0.33 nM | [36] |
| Electrochemical aptasensor based on poly(pyrrole-nitrilotriacetic acid)-aptamer film | 20 h | 100 min | 0.01 nM -1 μM | 0.01 nM | [37] |
| Aptamer-based electrochemical biosensor using dual-signaling strategy | 18 h | 60 min | 0.001 -1 nM | 0.41 pM | [38] |
| Electrochemical aptasensor based on gold nanoparticles dotted graphene modified glassy carbon electrode | 16 h | 30 min | 0.01 μM - 10 μM | 5 nM | [39] |
| Electrochemiluminescent aptasensor using $Mn^{2+}$-doped $NaYF_4$:Yb/Er upconversion nanoparticle | 34. 5 h | 30 min | 0.05 - 100 ng/mL | 0.037 ng/mL | [40] |
| Electrochemical aptasensor based on triple-signaling strategy | 20 h | 3 h | 1 pM- 100 pM | 0.19 pM | [41] |
| Electrochemical aptasensor using rationally engineered aptamer with MB label | 3 h | 5 min | 0.01-1000 μM | 0.01 μM | This work |

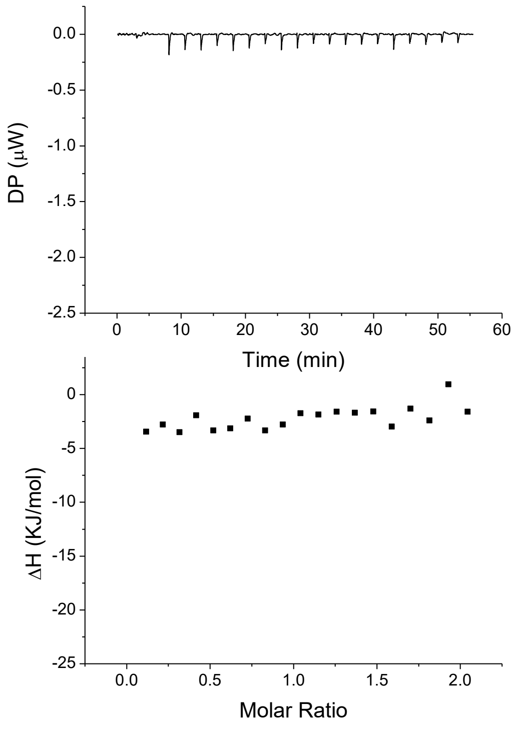


**Figure S1.** ITC measurement (top panel) and binding isotherm fitted by one set of the sites model (bottom panel) for the titration of BPA with aptamer BP63. During ITC analysis, 60 μL of BPA (200 μM) in injection syringe was titrated into 270 μL of aptamers (20 μM) in sample cell.

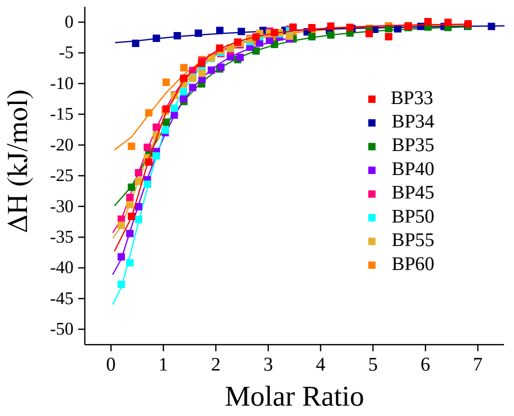


**Figure S2.** ITC analysis of binding affinities of BP60 and its truncated aptamers to BPA.

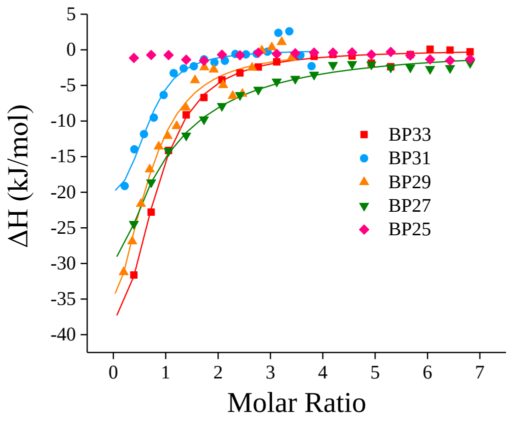


**Figure S3.** ITC analysis affinities of BP33 and truncated aptamers from BP33 to BPA.

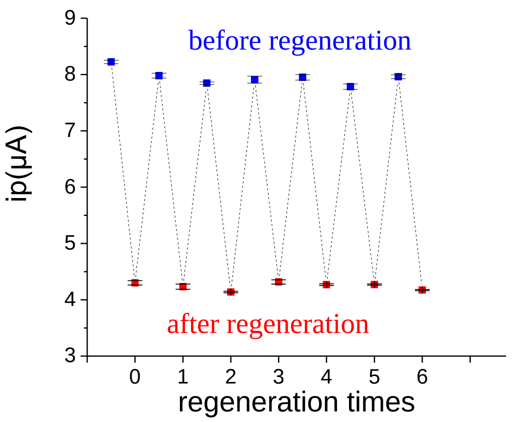


**Figure S4.** Effects of regeneration times on the peak current （ip）of the BP29-3'-MB electrode in SWV. After incubation with 500 μM BPA in the binding buffer containing 25 mM Tris-HCl (pH=8.0), 100 mM NaCl, 25 mM KCl and 10 mM $MgCl_2$, BP29-3'MB electrode was regenerated by rinsing with ultrapure water for 3 min. Frequency was set as 250 Hz.

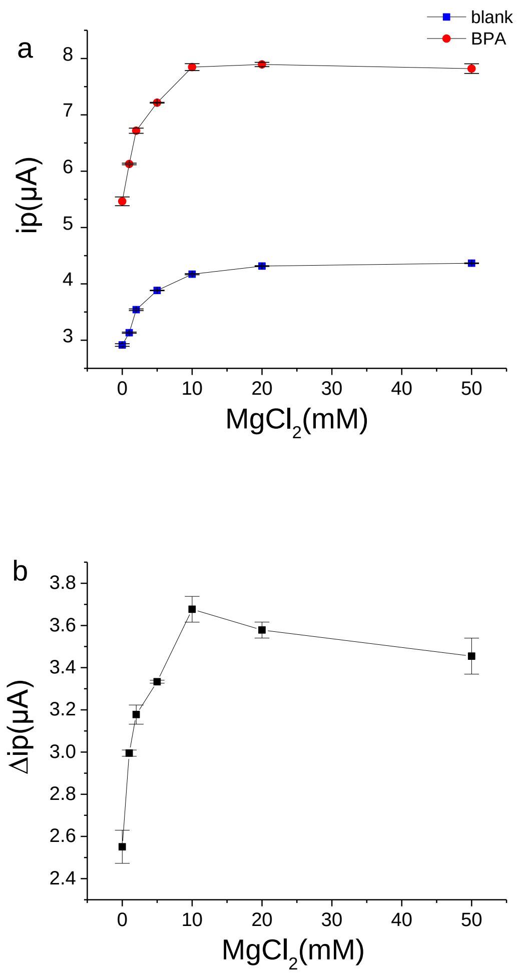


**Figure S5.** The effects of $MgCl_2$ on current signals of BP29-3'MB electrode in SWV in the absence and in the presence of BPA. (a) The peak current value of BP29-3'-MB electrode in the absence or the presence of 500 μM BPA. (b) The current signals change caused by 500 μM BPA. The binding buffer contained 25 mM Tris-HCl (pH=8.0), 100 mM NaCl, 25 mM KCl and varying concentrations of $MgCl_2$. Frequency of SWV was set as 250 Hz.

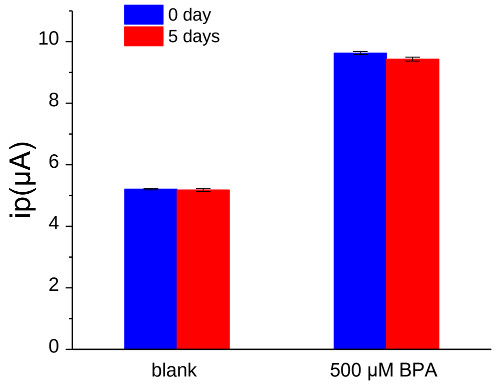


**Figure S6.** Stability test of responses of BP29-3'-MB modified electrode in SWV in the absence and in the presence of BPA after storage in binding buffer at 4 °C for 5 days.

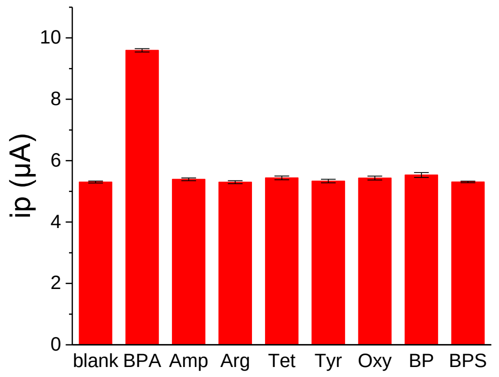


**Figure S7.** Selectivity of aptamer-based electrochemical switch assay for BPA detection. The concentrations of BPA, BP, BPS, Oxy, Try, Amp, Tet, and Arg were all 100 μM.

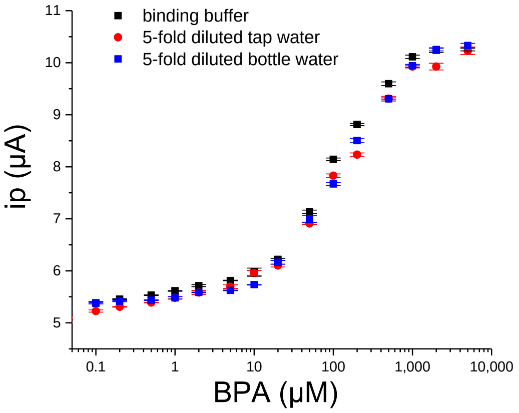


**Figure S8.** Aptamer-based electrochemical switch assay for detection of BPA spiked in 5-fold diluted tap water and 5-fold diluted bottle water.
